# Effects of Different Fertilizer Treatments, Environment and Varieties on the Yield-, Grain-, Flour-, and Dough-Related Traits and Cookie Quality of Weak-Gluten Wheat

**DOI:** 10.3390/plants11233370

**Published:** 2022-12-04

**Authors:** Hongya Wu, Zunjie Wang, Xiao Zhang, Junchan Wang, Wenjing Hu, Hui Wang, Derong Gao, Edword Souza, Shunhe Cheng

**Affiliations:** 1 Key Laboratory of Wheat Biology and Genetic Improvement for Low & Middle Yangtze Valley, Ministry of Agriculture and Rural Affairs, Lixiahe Institute of Agricultural Sciences, Yangzhou 225007, China; 2 Global Head Wheat Breeding, BASF Corporation, Beaver Crossing, NE 68313, USA

**Keywords:** weak-gluten wheat, fertilization, cookie quality, wheat quality

## Abstract

Weak-gluten wheat is the main raw material for crisp and soft foods such as cookies, cakes, and steamed breads in China. However, it remains challenging to find an appropriate fertilization regime to balance the yield and quality of wheat for special uses (such as cookie making). Here, four nitrogen (N) fertilizer treatments were compared in terms of effects on the yield-, grain-, flour-, and dough-related traits and cookie quality of nine weak-gluten wheat varieties. Compared with other treatments, the treatment M (which had 180 kg ha^−1^ N fertilizers with basal fertilizer:tillering fertilizer:jointing fertilizer = 5:1:4) was a superior fertilization strategy as it could ensure a higher yield (4.46 kg block^−1^) and proper traits related to cookie quality. Moreover, environmental conditions and wheat genotypes exhibited significant effects on many quality-related traits. The quality of Chinese crisp biscuits showed a significant association with unit weight, redness, and solvent retention capacity in lactic acid solution, while that of American cookies was influenced by thousand-grain weight, hardness, rate of yield flour, and formation time as indicated by the Mantel test. Additional Pearson correlation analysis demonstrated that thousand-grain weight, hardness, and rate of yield flour can affect the quality of American cookies. Our findings demonstrate that it is necessary to comprehensively consider local conditions, variety selection, and optimal fertilization to achieve high-quality weak-gluten wheat for cookie making.

## 1. Introduction

As one of the most widely planted and consumed crops, wheat can be used to produce numerous kinds of flour-derived products, such as biscuits, cakes, noodles, pasta, steamed buns, and breads [1]. The planting area of wheat in China is about 0.23 billion ha, accounting for nearly 13% of the wheat planting area worldwide [2]. The Chinese national standard “high-quality weak-gluten wheat” (GB/T17893-1999) stipulates that weak-gluten wheat should have the following properties: falling number (FN) ≥ 300 s; crude protein content (PC) ≤ 11.5% (dry basis); wet gluten content (WGC) of flour ≤ 22% (14% wet basis); stabilization time (ST) ≤ 2.5 min; and suitability for making cakes, crisp cookies and other foods [3]. 

Chinese weak-gluten wheat is similar to soft wheat, which has a soft grain texture and low protein content and is suitable for making biscuits, cakes, and other products [4,5]. The production of Chinese cookies from weak-gluten wheat has been predicted to dramatically increase from 1.05 million tons in 2004 to 14.4 million tons in 2024. Nevertheless, no more than 1.0% of the total weak-gluten wheat grain production can meet the requirement of cookie making for quality [6,7]. PC and WGC of flour are major parameters to evaluate the quality of wheat for special use. The gluten index can be used to simultaneously define the quantity and quality of gluten and is affected by different genotypes of wheat [8]. Wheat genotypes with low gluten content and weak-gluten network structure exhibit more easily spreading dough, which tends to have a larger cookie diameter during baking [9]. The content of grain protein, particularly seed storage protein, has a great impact on the quality of cookies by influencing the viscoelastic properties of wheat dough through the formation of a gluten network [9,10,11,12]. To obtain qualified wheat grains for cookie making, it is necessary to develop an excellent weak-gluten wheat variety or planting pattern to achieve both high yield and low grain PC. In actual planting practice, the reduction in nitrogen application rate usually causes a loss of grain yield, though it can decrease the grain PC [13,14,15]. Therefore, it is important to balance the grain PC and grain yield in weak-gluten wheat through some special cultivation measures. A decrease in nitrogen (N) application can significantly decrease both grain PC and grain yield; however, to some extent, the loss of grain yield can be compensated by increasing the top-dressed N ratio and plant density [7]. A previous study has revealed that increasing plant density, which is accompanied by an increase in effective ears, can improve the yield and dry matter accumulation while decreasing the grain PC of the soft wheat population [16].

Cookie quality is affected by protein and gluten content as well as by many features of wheat flour and dough. When assessing cookie diameter, water solvent retention capacity is a better parameter than farinograph or mixograph water absorption capacity and alveograph dough tenacity [17]. Falling number (FN) in wheat is an important quality predictor with a significant relation to α-amylase activityand therefore has a great impact on soft wheat end-use quality [18]. Soft wheat flour characteristics, such as damaged starch, amylose, and PC, were found to greatly affect the diameter of sugar snap cookies [19]. Moreover, several cookie quality parameters (hardness, diameter, thickness, and spread ratio) are influenced by soft wheat genotypes [20].

Although some studies have proposed some planting methods of soft or weak-gluten wheat to balance high yield and low PC [13,14,15], it remains undetermined whether these planting methods affect the physical and chemical properties of flour, the rheological properties of dough, and even the quality of cookies. Secondly, there have been rare reports about the differences in these traits among different weak-gluten wheat genotypes. Finally, the relationship between these traits and the quality of Chinese cookies or American cookies also needs to be evaluated in detail. In this study, we planted nine wheat varieties from the Middle and Lower Yangtze River Valleys in China in two experimental stations for two consecutive years with the same planting density and four widely used fertilization regimes to address the above issues. The results provide valuable information for the planting of weak-gluten wheat for cookie making and evaluating the effect of flour and dough properties on cookies.

## 2. Results

### 2.1. Differences in Grain, Flour, Dough, and Cookie Properties under Four Fertilizer Treatments

**Figure 1:**
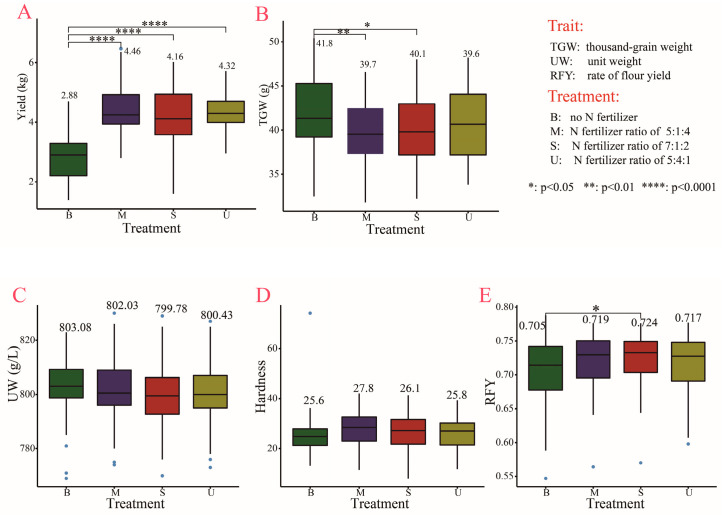
Yield- and grain-related traits under the four fertilizer treatments.(**A**–**E**) Comparison of four fertilizer treatments in yield (**A**); thousand-grain weight (**B**); unit weight (**C**); hardness (**D**); and rate of flour yield (**E**). Box diagrams present the data in the inter-quartile ranges. The lines across the boxes, whiskers, and blue circles denote the median values, the 10th and 90th percentiles and outliers, respectively.

Among the four fertilizer treatments, the M treatment (i.e., the N fertilizer ratio of base fertilizer:tiller fertilizer:jointing fertilizer is 5:1:4) had the highest yield (4.46 kg block^−1^), followed by U (i.e., the N fertilizer ratio of base fertilizer:tiller fertilizer:jointing fertilizer is 5:4:1) and S (i.e., the N fertilizer ratio of base fertilizer:tiller fertilizer:jointing fertilizer is 7:1:2), which had 4.32 kg block^−1^ and 4.16 kg block^−1^ of yield, respectively. B (control, i.e., no fertilizer application) showed a significantly lower yield than the other three fertilizer treatments (Figure 1A) but the highest thousand-grain weight (TGW) (41.8 g), which was significantly different from that of M (39.7 g) and S (40.1 g) (Figure 1B). These results suggested that the increase in yield might not result from an increase in TGW. Both unit weight (UW) and hardness of wheat grains showed no significant difference in each pairwise comparison of the four fertilizer treatments (Figure 1C,D). The three fertilizer treatments (M, S, and U) exhibited higher rate of flour yield (RFY) levels relative to the control (B), though both M and U were not significantly different from B (Figure 1E).

In terms of physical and chemical properties, M, S, and U exhibited higher PC, WGC, and SDS sedimentation value (SSV) than B (Table 1). M had a PC of 11.6%, which reached the criterion of medium-gluten wheat (>11.5%). This could be attributed to the application of nitrogen fertilizer, because no application of nitrogen fertilizer can lead to less accumulation of gliadin and low-molecular-weight glutenin. No significant difference in both FN and whiteness (including brightness (L*), redness (a*) and yellowness (b*)) was found in each pairwise comparison of the four treatments (Table 1).

In terms of rheological properties of wheat dough, no significant difference was observed in formation time (FT), ST, peak viscosity (PV), and breakdown value (BV) among the four groups, except for the significantly higher value of the blowing instrument parameter (P/L) in B (1.29) than that in the other three fertilizer treatments (Table 1). One possible explanation is that treatment B involved no application of fertilizers, which might greatly reduce the accumulation of gliadin and low-molecular-weight glutenin, leading to a decrease in L value and an increase in P/L value.

Among the four properties of solvent retention, only solvent retention in lactic acid solution (LAS) was lower in B (91.33), which was significantly different from that under the other three treatments (Table 1). This result indicated that treatment B resulted in poorer properties of soft gluten, and there was no significant difference in the other three properties in each pairwise comparison of the four treatments (Table 1).

**Figure 2:**
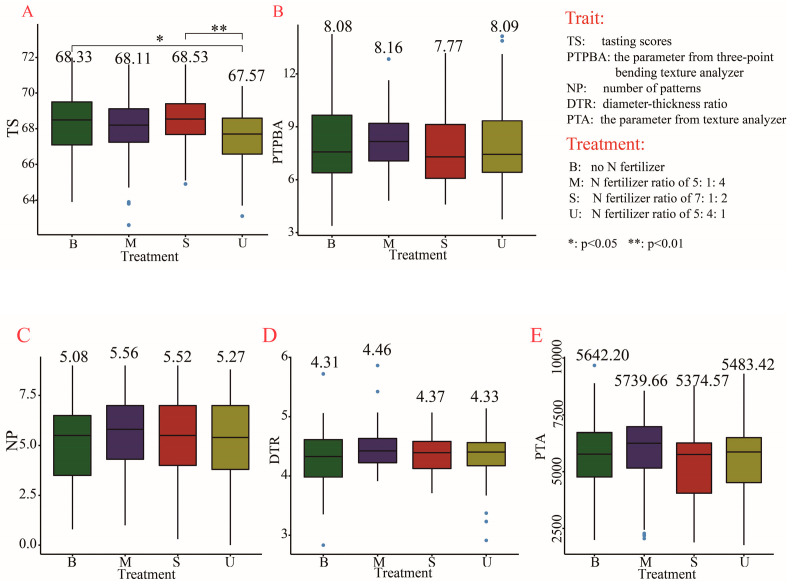
Quality evaluation of Chinese crisp biscuits and American cookies under four fertilizer treatments. (**A**–**E**) Comparison among four fertilizer treatments in tasting score (**A**); the parameter from three-point bending texture analyzer (**B**); the number of patterns (**C**); diameter–thickness ratio; (**D**) and the parameter from texture analyzer (**E**). Box diagrams present the data in the inter-quartile ranges. The lines across the boxes, whiskers, and blue circles denote the median values, the 10th and 90th percentiles and outliers, respectively. The number above the box represents the average level of the trait in each treatment.

Tasting scores (TS) and the parameter from three-point bending texture analyzer (PTPBA) were used to evaluate the quality of Chinese crisp biscuits, while the number of patterns (NP), diameter–thickness ratio (DTR), and the parameter from the texture analyzer (PTA) were used to evaluate American cookies. As a result, TS in U was the lowest (67.75) and showed significant differences from that of B (68.33) and S (68.53), respectively (Figure 2A). However, no significant difference was found in PTPBA, NP, DTR, and PTA in each pairwise comparison of the four treatments. These results suggested that fertilizer treatments could affect the taste of Chinese crisp biscuits but do not influence the quality of American cookies.

### 2.2. Comparison of Grain, Flour, and Cookie Properties in Different Growth Environments

**Figure 3:**
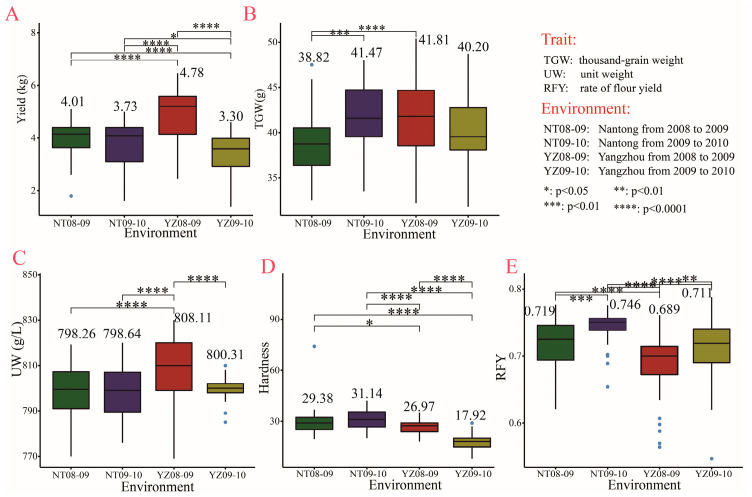
Yield and grain related traits in four growth environments.(**A**–**E**) Comparison among four growth environments in yield (**A**); thousand-grain weight (**B**); unit weight (**C**); hardness (**D**) and rate of flour yield (**E**). Box diagrams present the data in the interquartile ranges. The lines across boxes, whiskers, and blue circles denote the median values, the 10th and 90th percentiles, and outliers, respectively. The number above the box represents the average level of trait in each treatment.

The experiments were carried out in four growth environments: Yangzhou experimental station in 2008–2009 (YZ08-09), Yangzhou experimental station in 2009–2010 (YZ09-10), Nantong experimental station in 2008–2009 (NT08-09), and Nantong experimental station in 2009–2010 (NT09-10). Yield and four grain-related traits (TGW, UW, hardness, and RFY) showed significant differences in at least two of the four growth environments (Figure 3A–E), suggesting that the growth environment has a great influence on these traits. In the four growth environments, YZ08-09 had the highest yield (4.78 kg), TGW (41.81 g), and UW (808.11 g L^−1^), but the lowest RFY (0.689) (Figure 3A–C,E).

NT08-09 and NT09-10 showed higher values of PC, WGC, SSV, FN, a*, b*, ST, PV, and LAS, but lower values of L* than YZ08-09 and YZ09-10 (Table 1). No significant differences in FT and DW were observed in any pairwise comparison of the four growth environments (Table 1). For other dough rheological properties (BV and P/L) and solvent retention properties (SS and SCS), there were great differences between different years in the same station (Table 1), indicating that these properties are easily affected by climatic conditions.

**Figure 4:**
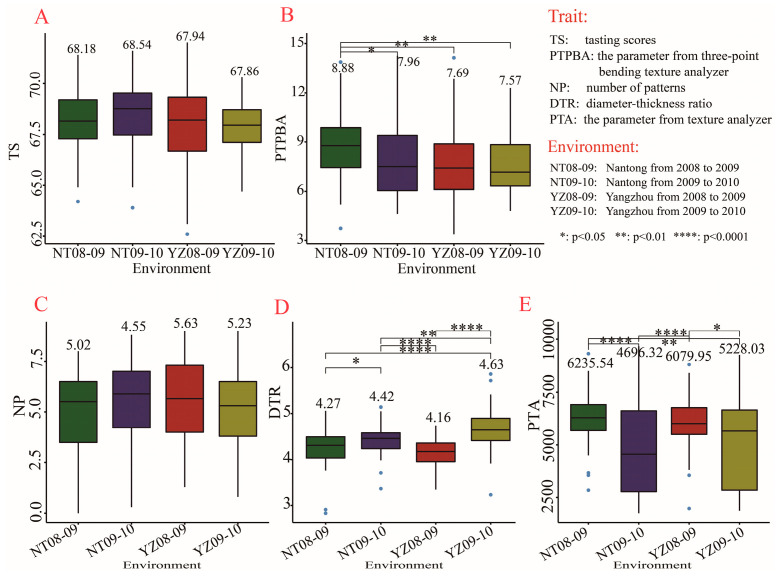
Quality evaluation of Chinese crisp biscuits and American cookies in four growth environments. (**A**–**E**) Comparison among four growth environments in tasting score (**A**); parameter from three-point bending texture analyzer (**B**); number of patterns (**C**); diameter–thickness ratio (**D**) and parameter of texture analyzer (**E**). Box diagrams present the data in the interquartile ranges. The lines across the boxes, whiskers, and blue circles denote the median values, the 10th and 90th percentiles, and outliers, respectively. The number above the box represents the average level of trait in each environment.

For Chinese crisp biscuits, it was also found that compared with Yangzhou experimental station, Nantong experimental station had higher levels of TS and PTPBA for two consecutive years, though the difference in TS was not significant (Figure 4A,B). Different growth environments showed no effect on the NP of American cookies but had a great impact on DTR and PTA (Figure 4C–E). The DTR in Nantong and Yangzhou experimental stations was 4.27 and 4.16 in 2008–2009, respectively, which were significantly lower than those in 2009–2010 (Figure 4D). However, the PBA in Nantong and Yangzhou experimental stations in 2008–2009 was 6235.54 and 6079.95, respectively, which were significantly higher than those in 2009–2010 (Figure 4E).

### 2.3. Comparison of Grain, Flour, and Cookie Properties among Varieties

**Figure 5:**
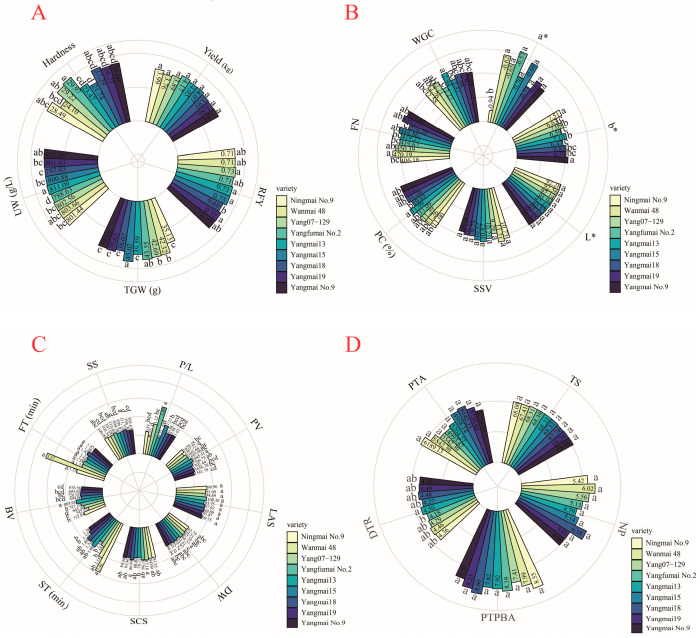
Traits of the nine tested weak-gluten wheat varieties. (**A**–**D**) Comparison among nine wheat varieties in yield- and grain-related traits: (**A**) including yield, TGW, UW, hardness, and RFY), physical and chemical properties (**B**); including PC, WGC, SSV, FN, L*, a*, and b*), rheological properties of dough, and solvent retention (**C**); including FT, ST, PV, BV, P/L, DW, SS, SCS, and LAS) and properties of Chinese crisp biscuits and American cookies (**D**); including TS and PTPBA for Chinese crisp biscuits, and NP, DTR, and PBA for American cookies). TGW, thousand-grain weight; UW, unit weight; RFY, rate of flour yield; PC, protein content; WGC, wet gluten content; SSV, SDS sedimentation value; FN, falling number; L*: brightness; a*: redness; b*: yellowness; FT, formation time; ST, stabilization time; PV, peak viscosity; BV, breakdown value; P/L, blowing instrument parameter. DW, SS, SCS, and LAS denote retention capacity of flour with de-ionized water (DW), sucrose solution (SS), sodium carbonate solution (SCS), and lactic acid solution (LAS) as the solvent, respectively. TS, tasting score; PTPBA, the parameter from the three-point bending texture analyzer; NP, number of patterns; DTR, diameter-thickness ratio; PTA, the parameter from the Texture Analyzer. Different lowercase letters indicate the significance of the difference.

Nine weak-gluten wheat varieties were tested in this study, and they showed no difference in yield (Figure 5A). One possible reason is that the yield difference among varieties was diluted by different fertilizer treatments or different growth environments, and the other possible reason is that the parents of the nine varieties have relatively similar genetic backgrounds. Among the nine varieties, Yangmai 15 had the highest TGW (45.02 g), the lowest hardness (21.74 g L^−1^), the second highest yield (4.07 kg block^−1^), and the highest RFY (0.73) (Figure 5A). Moreover, Yangmai 15 exhibited the lowest PC (10.65%), the second lowest WGC (2.74), the highest flour whiteness L* value (92.57), the lowest b* value (6.74), and the third highest FN (444.86) (Figure 5B). However, Yangmai 15 was found to have medium values in the rheological properties of dough, solvent retention (Figure 5C), and the parameters of Chinese crisp biscuits and American cookies (Figure 5D).

In the five properties of Chinese crisp biscuits and American cookies, no property showed a significant difference between varieties except for DTR (Figure 5D). However, Yangmai 13 might be more suitable for making American cookies because it showed the highest DTR (4.50) and the lowest NP (4.79), though its NP showed no significant difference from that of other varieties (Figure 5D). In addition, Yangmai 13 exhibited the highest RFY (0.73), the highest UW (811.09 g L^−1^), the second lowest hardness (23.47), the third highest yield (4.05 kg block^−1^), the highest WGC (2.94), the highest PC (11.99%), the second highest SSV (10.82), the second lowest FT (1.29 min), the third highest ST (1.88 min), the second lowest SS (104.70), the second lowest P/L (0.71), the highest PV (1845.31), the lowest LAS (93.75), the lowest DW (63.31), the lowest SCS (79.77), and the highest BV (883.91) (Figure 5A–C).

### 2.4. Correlation Analysis and Mantel Test

**Figure 6:**
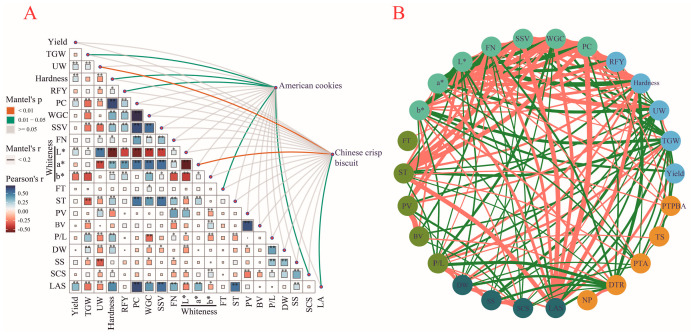
Pearson correlation coefficients of traits and Mantel test between cookie quality and traits. (**A**) Mantel test plot. The ** and * in the upper triangle denote the significance level of 0.01 and 0.05, respectively. (**B**) Correlation network diagram among traits. The red and green lines represent significant positive correlations and significant negative correlations at the level of 0.05, respectively. The thickness of the line represents the size of the correlation coefficient between the two traits. TGW, thousand-grain weight; UW, unit weight; RFY, rate of flour yield; PC, protein content; WGC, wet gluten content; SSV, SDS sedimentation value; FN, falling number; L*: brightness; a*: redness; b*: yellowness; FT, formation time; ST, stabilization time; PV, peak viscosity; BV, breakdown value; P/L, blowing instrument parameter. DW, SS, SCS, and LAS denoted the retention capacity of flour with de-ionized water (DW), sucrose solution (SS), sodium carbonate solution (SCS), and lactic acid solution (LAS) as the solvent, respectively. TS, tasting score; PTPBA, the parameter from the three-point bending texture analyzer; NP, number of patterns; DTR, diameter-thickness ratio; PTA, the parameter from the Texture Analyzer.

Several yield- and grain-related traits were significantly correlated with each other, as well as with some flour physical and chemical properties, dough rheological properties, and solvent retention properties (Figure 6A,B). The yield was significantly positively correlated with UW, hardness, PC, L*, and LAS but negatively correlated with b* at the 0.01 level (Figure 6A,B). TGW was found to have significant positive correlations with UW (*p* < 0.01), L* (*p* < 0.05), P/L (*p* < 0.01), and DW (*p* < 0.01) (Figure 6A,B) but exhibited significant negative correlations (*p* < 0.01) with PC, WGC, SSV, b*, ST, and BV, SS, and LAS (Figure 6A,B). RFY was found to be significantly positively correlated with hardness, PC, WGC, SSV, a*, and b* but significantly negatively correlated with UW, L*, and PV (Figure 6A,B). PC had significant correlations with TGW, hardness, RFY, WGC, SSV, FN, L*, a*, ST, and LAS (Figure 6A,B). WGC was also found to have significant correlations with TGW, hardness, RFY, SDS, L*, a*, ST, and LAS (Figure 6A,B), suggesting that these traits might have great impacts on both PC and WGC. FT was found to have no significant correlation with other traits except for WGC (Figure 6A,B), implying that FT is independent of other traits.

The Mantel test was performed to investigate the relationship between the traits and quality of Chinese crisp biscuits or American cookies. As a result, the quality of American cookies showed a significant relationship with TGW, hardness, RFY, and FT at the Mantel *p*-value of 0.01–0.05 (Figure 6A), suggesting that these traits might have important impacts on the quality of American cookies (Figure 6A). However, the quality of Chinese crisp biscuits showed a significant relationship with UW (Mantel *p*-value < 0.01), whiteness a* (mantel *p* value < 0.01), and LA (mantel *p*-value: 0.01–0.05) (Figure 6A), implying that the quality of Chinese biscuits is affected by UW, whiteness a* and LA. Moreover, among the three properties of American cookies, both DTR and PBA showed a negative correlation with TGW (Figure 6B). Hardness showed a significant negative correlation with DTR (Figure 6B), while RFY displayed a significant negative correlation with PTA. These findings indicated that TGW, hardness, and RFY could affect the quality of American cookies.

## 3. Discussion

### 3.1. M Treatmentis a Superior Fertilization Mode

Nitrogen is an essential element of wheat growth and grain quality [21]. There have been numerous studies of suitable fertilization (particularly N fertilizer) for high-yield and high-quality of medium- or strong-gluten wheat varieties [1,22,23]. Relatively consistent conclusions have been drawn: increasing N fertilizer or transferring N fertilizer to the later growth stage can simultaneously improve the yield, quality, and grain PC of strong-gluten wheat [22,24]. A recent study has suggested that moderately high N fertilizer application under drought environment can improve gluten accumulation [24]. Under the total N fertilizer application of 195 kg ha^−1^ (105 kg ha^−1^ base fertilizer and 90 kg ha^−1^ topdressing fertilizer), both grain yield and PC of micro-sprinkling irrigation could be increased relative to those in conventional irrigation practice [25]. However, weak-gluten wheat requires appropriate fertilization measures to ensure the yield so that the varieties can meet the requirement of special uses. Inconsistent conclusions have been made in different studies of fertilization (especially N fertilizer) for weak-gluten wheat [13,14,15]. Under 150 Kg N ha^−1^ with 50% top-dressed N and 360 × 10^4^ plantsha^−1^, the grain yield loss of soft wheat can be compensated, while PC can be decreased (Zheng et al. 2022) [7]. The result of the fertilization mode might be attributed to the downregulation of high-molecular-weight genes and low-molecular-weight genes in mature grains [7]. A previous study concluded that the quality of weak-gluten wheat can reach the national standard of China when the ratio of basal fertilizer to topdressing fertilizer is 5:5 [26]. Zhu et al. [27] reported that both high yield and weak gluten properties could be achieved when the rate of basal fertilizer:tillering fertilizer:elongation N fertilizer was 7:1:2 with 180 kg N ha^−1^ and 2.4 million seedlings. Li et al. [28] studied the effect of two fertilizer treatments on Ningmai No.9 at a basic seedling density of 2.4 million ha^−1^. In the two fertilizer treatments, the treatment with a basal fertilizer:tiller fertilizer:two-leaf fertilizer ratio of 7:1:2 showed more appropriate C and N content and C/N ratio than the treatment with a ratio of 5:1:4, indicating that the ratio of 7:1:2 could more likely achieve high yield and high quality of weak-gluten wheat.

Our results about yield suggested that the M treatment (the ratio of base fertilizer:tiller fertilizer:jointing fertilizer is 5:1:4) is a superior fertilization mode (Figure 1A), because the M treatment had a higher yield than the B treatment (control) and even than that under the above mentioned optimal ratio of 7:1:2 (Figure 1A). One study has indicated that a lack of N fertilizer in the sowing–tillering period may result in limited N availability for wheat uptake in the subsequent period as in the tillering–first-node period [29]. Another study suggested that a high rate of N fertilizer can accelerate the expansion of leaf area, especially before stem elongation, and result in high pre-anthesis leaf area duration [30]. This may explain the result that an increase in the proportion of jointing fertilizer in treatment M led to a higher yield (Figure 1A). It was reported that 225 kg ha^−1^ N application and a base fertilizer: top-dressing fertilizer ratio of 6: 4 contributed to the highest yield of the Jintai 182 variety [31]. We found that the increase in yield under the M treatment might not be due to the increase in TGW (Figure 1B) but may be related to the increase in the number of ears and grains per ear [31]. Further experiments should be carried out to explore the effect of M treatment on yield-related traits and verify the above speculation.

From the perspective of physical and chemical properties such as PC, WGC, SSV, FT, and ST, non-fertilizer treatment (B) resulted in closer values to the standards of high-quality weak-gluten wheat than other fertilizer treatments (M, U, and S) (Table 1) [2]. However, the non-fertilizer treatment led to a higher P/L value than fertilizer treatments, which might be ascribed to the relatively more significant reduction in both gliadin and low-molecular-weight gluten caused by the lack of N fertilizer. It has been reported that the lack of N fertilizer could decrease the L value and increase the P/L value. Although non-fertilizer treatment seemed to optimize part of the physical and chemical quality properties, the increase in dough elasticity and ductility (increased P/L value) is not conducive to the making of high-quality cookies (Table 1). The highest P/L value in B and the significant negative correlation between P/L and DTR to some extent can explain the lowest NP and DTR in American cookies under treatment B (Table 1; Figure 6B). As a matter of fact, some agronomic practices such as sowing time and amount of N fertilizer have significant impacts on biscuit wheat quality, such as SDS sedimentation value, protein content, and wet and dry gluten content [32]. Therefore, some lines and varieties that are less responsive to extra N should be selected as parents in the breeding of weak gluten wheat for better biscuit quality [32].

### 3.2. Two Types of Biscuits Are Affected by Different Traits

Some characteristics of grains, flour, and dough can affect the quality of biscuits. It has been reported that cookie-baking quality is largely influenced by grain PC (especially for seed storage proteins) [9,10,11,12,33]. Gluten proteins can form a proteinaceous network around the starch granules during the senesce of the starchy endospermcells [34]. Gaines et al. [35] and Chen [36] proposed that there is a significant negative correlation between PC and biscuit quality, which is consistent with the negative correlation between DTR and PC found in this study (Figure 6B). Zhang et al. [37] also reported that there is a low correlation coefficient between grain protein and biscuit diameter. In addition, the gluten content and quality of flour have more obvious effects on dough traits than protein. During baking, wheat dough with lower gluten content and weaker gluten network structure is easier to spread and tends to produce crispier biscuits with larger diameters [9]. Chen [38], Bai and Lin [39], and Lai et al. [40] demonstrated that gluten content is significantly negatively correlated with biscuit quality, but our results showed no correlation between the WGC and the biscuit quality. The national standard of China (GB/T17893-1999) pays more attention to the ST (≤2.5 min) of weak-gluten wheat [3]. Moreover, some studies have revealed that the FT is closely related to the quality of biscuits. The quality of soft wheat cakes and biscuits has significant negative correlations with the FT and water absorption as measured by Farinograph but has no correlation with the ST [38,39]. We suspect that the quality of American cookies might be influenced by FT according to the Mantel test, though FT showed no correlation with other traits (Figure 6A,B). Moreover, TGW, hardness, and RFY might affect the quality of American cookies (Figure 6A). Yamamoto et al. [41] and Zhang [42] reported that compared with traits determined by Farinograph and extensometer, those determined by a blowing instrument (P/L) can better explain the variations in biscuit quality. We also found that P/L had significant correlations simultaneously with DTR and TS (Figure 6A), suggesting that P/L could affect the quality of both types of biscuits. Similar results were obtained by this study and that of Gaines [43], i.e., the diameter of biscuits was negatively correlated with the four solvent retention properties (DW, SS, SCS, LAS) (Figure 6B). Guttieri et al. [44] studied 26 soft spring wheat varieties planted in seven sites and found that the solvent retention capacity (SRC) method could effectively evaluate the difference between genotype and environment. In our study, three solvent retention properties (SS, SCS, and LAS) besides DW showed significant differences in at least one pairwise comparison of environments (Table 1). Guttieri and Souza [45] studied the recombinant inbred populations from three different soft wheat combinations. Their results showed that the genotype variance of the three populations accounted for 67–90% of the total variance, indicating that genotype is the main factor affecting the SRC quality indicators. We also found that genotype might play another important role besides the environment in explaining the variation in SRC because the nine varieties showed differences in all four traits except for LA (Table 1). Zhang [42] also reported that water, lactic acid, sodium carbonate, and sucrose SRC are significantly negatively correlated with biscuit diameter, which could explain 60.84%, 70.56%, 62.41%, and 64.00% of the variance in biscuit diameter, respectively. The correlation analysis results showed that these four SRC traits can affect the quality of American biscuits with negative correlations (Figure 6B). To sum up, the quality of Chinese crisp biscuits might be influenced by solvent retention capacity in lactic acid solution, while that of American cookies is affected by thousand-grain weight, hardness, rate of yield flour, and formation time.

## 4. Conclusions

In this study, the M treatment (the ratio of base fertilizer:tiller fertilizer:jointing fertilizer is 5:1:4) was identified as a superior fertilization pattern. Moreover, environmental conditions and wheat genotype exhibited significant effects on many quality traits. The quality of Chinese crisp biscuits might be influenced by solvent retention capacity in lactic acid solution, while that of American cookies was influenced by thousand-grain weight, hardness, rate of yield flour, and formation time. These findings can provide guidance for the planting of weak-gluten wheat for cookie making and evaluating the effect of flour and dough properties on cookies. In the future, in order to obtain higher yield and better quality traits related with biscuit quality, the M treatment should be further studied in combination with more measures, such as different planting densities.

## 5. Materials and Methods

### 5.1. Plant Materials

Nine weak-gluten wheat varieties were selected from the Middle and Lower reaches of the Yangtze River, the main weak-gluten-wheat-producing area in China (Table 2). All nine varieties were bred in the Middle and Lower Yangtze River Valleys and have strong adaptability to the temperature, light, moisture, and soil environment of the region. These varieties were approved from 1996 to 2008 (Table 2). Among them, Yangmai 15 has the strongest adaptability because it has been approved to be cultivated in other provinces in China besides the province where it was released (Table 2).

### 5.2. Experimental Design

#### 5.2.1. Experimental Stations

The experiments were conducted in the experimental fields of the Lixiahe Institute of Agricultural Sciences (located in Yangzhou city, Jiangsu province, China) and crop cultivation base in Hai’an county (located in Nantong city, Jiangsu province, China). The experiments were carried out for two consecutive wheat growth periods from November 2008 to June 2009 and from November 2009 to June 2010. The four growing environments were hereafter referred to as YZ08-09 (Yangzhou experimental fields from 2008 to 2009), YZ09-10 (Yangzhou experimental fields from 2009 to 2010), NT08-09 (Nantong experimental fields from 2008 to 2009), and NT09-10 (Nantong experimental fields from 2009 to 2010).

#### 5.2.2. Block Design and Fertilizer Treatments

In both experimental stations, the soil was sandy and the preceding crop was rice before the planting of wheat. The soil nutrient status of the two experimental stations was similar. The content of organic matter in Yangzhou and Nantong experimental fields was 11.62 and 10.59 mg g^−1^, respectively. Soil-available N, P, and K in the Yangzhou experimental fields were 41.27, 23.92, and 139.81 mg kg^−1^, respectively, while those in the Nantong experimental fields were 44.06, 27.23, and 146.98 mg kg^−1^, respectively. The average maximum temperature, average minimum temperature, average daily temperature, and average monthly rainfall of wheat growing season (from January to June) in Yangzhou were 28.25 °C, −0.75 °C, 13.25 °C, and 37.42 mm, respectively, while these were 27.00 °C, −0.75 °C, 12.50 °C, and 40.62, respectively, in Nantong. The experimental fields at each station were divided into multiple blocks (3 m × 2.22 m). Except for the control blocks, each block was applied with 0.12 kg N fertilizers in total, which was equivalent to 180 kg ha^−1^N fertilizers. Four fertilizer treatments were applied to different blocks, including (1) control or blank (i.e., no N fertilizer application); (2) treatment similar to the high-yield planting method (i.e., the N fertilizer ratio of base fertilizer:tiller fertilizer:jointing fertilizer is 5:1:4); (3) widely used fertilization method for weak-gluten wheat (i.e., the N fertilizer ratio of base fertilizer:tiller fertilizer:jointing fertilizer is 7:1:2), and (4) soft wheat fertilization method commonly used in the USA (i.e., the N fertilizer ratio of base fertilizer:tiller fertilizer:jointing fertilizer is 5:4:1). Hereafter, the treatments from (1) to (4) are represented by B, M, S, and U, respectively. For all plots, 120 kg P_2_O_5_ ha^−1^ and 120 kg K_2_O ha^−1^ were applied before sowing as the basal fertilizer. These fertilization treatments at each experimental station were repeated twice in each wheat growth period with a completely randomized block design. After emergence, seedling thinning was carried out for each block so that the density of seedlings was 1500 plants per block.

### 5.3. Trait Measurement

#### 5.3.1. Yield and Grain-Related Traits

Wheat grains in each block were harvested and naturally dried, followed by the measurement of yield, TGW, UW, and hardness. UW was measured with an HGT-1000 Unit Weight Measuring Instrument according to the national grain standard GB1351-78 of China [46]. Hardness was measured by a Single Grain Characteristic Test Instrument. Harvested grains in each block were made into flour, and then RFY was calculated with the following formula:

RFY = (Flour Yield)/(Grain Yield).

#### 5.3.2. Physical and Chemical Properties

PC was determined with the Kjeldahl determination method according to the standard GB2905-82 of China [47]. WGC was determined using the 2200 type Gluten Measuring Instrument according to the standard GB/T14608-93 of China [48]. SSV was measured using56-61A of the AACC method. FN was quantified using an 1800-type FN Instrument (Falling Number Company, Germany). The whiteness of flour, including brightness (L*), yellowness (b*), and redness (a*), was measured using a Minolta CR-310 chromometer.

#### 5.3.3. Rheological Properties of Dough

Both FT and ST were measured using a Farinograph (Brabender Company, Duisburg, Germany) with the AACC54-21 method. Both PV and BV were quantified using an RVA-super3 (Newport Scientific Company, Berlin, Germany). P/L was measured using an Alveograph (Chopin Company, Marseille France) according to AACC54-21.

#### 5.3.4. Solvent Retention Capacity

The retention capacity of flour in four solvents was measured according to AACC54-11 [49]. Four retention capacities included de-ionized water (DW), 50% (*w*/*w*) sucrose solution (SS), 5% (*w*/*w*) sodium carbonate solution (SCS), and 5% (*w*/*w*) lactic acid solution (LAS). Detailed experimental steps are presented in the Supplementary Materials and methods.

#### 5.3.5. Evaluation of Two Types of Cookies

Two types of cookies, Chinese crisp biscuits and American cookies, were made according to standard SB/T10141-93 [50] of China and standard AACC10-52 of America [51], respectively. Detailed steps are presented in the Supplementary Materials and methods. TS of Chinese crisp biscuits was evaluated as an average of the scores (percentage system) from seven experts with evaluation experiences. Furthermore, PTPBA was used to evaluate Chinese crisp biscuits. American cookies were evaluated by numbers of NP, DTR, and the parameter from PTA. DTR was measured according to AACC10-52.

### 5.4. Statistical Analysis

The non-parametric Kruskal–Wallis H test was performed to evaluate the differences among four fertilizer treatments, among four growth environments and nine varieties in all traits, because all traits did not conform to both normal distribution and variance homogeneity test. Kruskal–Wallis H test, normal distribution test, and variance homogeneity test were performed in R with functions kruskal_test, shapiro_test and, levene_test, respectively. The box diagram and radial ring diagram were drawn in R with the ggplot2 package. Correlation analysis (Pearson correlation) and Mantel test were carried out by cor and mantel_test functions in R, respectively, and the results were visualized through the ggplot2, linkET, and igraph packages in R.

## Figures and Tables

**Table 1:** Physical and chemical properties of flour, rheological properties of dough, and solvent retention of wheat under different fertilizer treatments and in different growth environments.

Treatments	Physical and Chemical Properties of Flour							Rheological Properties of Dough					Solvent Retention			
	PC (%)	WGC (%)	SSV	FN	Whiteness			FT (min)	ST (min)	PV	BV	P/L	DW	SS	SCS	LAS
					L*	a*	b*									
B	10.6 b	2.45 b	8.54 b	429.95 a	92.4 a	−0.735 a	6.93 a	1.32 a	1.47 a	1628.63 a	694.07 a	1.29 a	65.88 a	105.83 a	83.43 a	91.33 b
M	11.6 a	2.71 a	11.1 a	427.99 a	92.3 a	−0.647 a	6.98 a	1.44 a	1.89 a	1625.38 a	699.04 a	1.00 b	65.52 a	107.63 a	83.00 a	100.00 a
S	11.4 a	2.64 ab	10.8 a	430.26 a	92.3 a	−0.694 a	6.82 a	2.13 a	1.95 a	1677.39 a	720.21 a	0.91 b	65.71 a	108.02 a	84.94 a	99.57 a
U	11.4 a	2.68 a	10.3 a	430.92 a	92.3 a	−0.663 a	6.87 a	1.46 a	1.96 a	1660.07 a	717.90 a	0.91 b	66.02 a	107.58 a	93.91 a	98.44 a
**Environment**																
NT08-09	12.4 a	2.81 ab	12.2 a	490.05 a	92.1 c	−0.593 b	6.66 b	1.52 a	3.12 a	1734.61 a	727.94 a	1.20 a	66.02 a	108.04 a	82.05 b	105.63 a
NT09-10	12.0 b	2.97 a	11.9 ab	414.94 bc	91.5 d	−0.456 a	7.26 a	2.26 a	1.72 b	1594.13 b	719.51 a	1.00 ab	66.12 a	105.12 b	84.77 ab	98.90 b
YZ08-09	10.7 c	2.28 c	7.57 d	421.17 b	92.9 a	−0.943 d	6.54 b	1.31 a	1.24 b	1592.89 b	659.50 b	1.09 a	66.18 a	107.33 ab	82.64 ab	95.23 b
YZ09-10	9.83 d	2.42 c	9.04 c	392.96 c	92.7 b	−0.747 c	7.13 a	1.26 a	1.20 b	1669.83 ab	724.26 a	0.82 b	64.81 a	108.58 a	85.83 a	89.59 c

Note: PC, protein content; WGC, wet gluten content; SSV, SDS sedimentation value; FN, falling number; L*: brightness; a*: redness; b*: yellowness; FT, formation time; ST, stabilization time; PV, peak viscosity; BV, breakdown value; P/L, blowing instrument parameter. DW, SS, SCS, and LAS denote retention capacity of flour with de-ionized water (DW), sucrose solution (SS), sodium carbonate solution (SCS), and lactic acid solution (LAS) as solvent, respectively. B, no fertilizer was applied; M, proportion of base fertilizer:tiller fertilizer:jointing fertilizer is 5:1:4; S, the ratio of base fertilizer:tiller fertilizer:jointing fertilizer is 7:1:2; U, the ratio of base fertilizer:tiller fertilizer:jointing fertilizer is 5:4:1. Different lowercase letters indicate the significance of the difference.

**Table 2:** Information of the nine weak-gluten wheat varieties.

Name	Breeding Institutions	Sources	Years
Ningmai No.9	Lixiahe Institute of Agricultural Sciences	Yangmai No.6/Xifeng	1997
Wanmai 48	Anhui Agricultural University	Aizao781/Wansu8802	2002
Yang07-129	Lixiahe Institute of Agricultural Sciences	Yang89-40/Yangmai No.158	2007
Yangfumai No.2	Lixiahe Institute of Agricultural Sciences	Yangfumai 1-9012/Yangmai No.158	2002
Yangmai13	Lixiahe Institute of Agricultural Sciences	Yang88-84/(MarisDove/Yangmai No.3)	2003
Yangmai15	Lixiahe Institute of Agricultural Sciences	Yang89-40(Yangmai No.4/Yang80)/Chuanyu21526	2004
Yangmai18	Lixiahe Institute of Agricultural Sciences	Ning94/3/Yang1586/2/88-128/NannongP045	2008
Yangmai19	Lixiahe Institute of Agricultural Sciences	1583/3/(Y.C./Yang5)F1/2/Yang85-584	2008
Yangmai No.9	Lixiahe Institute of Agricultural Sciences	Jiansan/Yangmai No.5	1996

## Data Availability

Data will be made available on request.

## Supplementary Materials

The following supporting information can be downloaded at: https://www.mdpi.com/article/10.3390/plants11233370/s1. Reference [49,50,51] is cited in the supplementary materials
