# Supplementary material for: Effects of Different Fertilizer Treatments, Environment and Varieties on the Yield-, Grain-, Flour-, and Dough-Related Traits and Cookie Quality of Weak-Gluten Wheat
Source: Plants (Basel). 2022 Dec 4;11(23):3370. doi: 10.3390/plants11233370 (PMC9738188; doi:10.3390/plants11233370)
Supplement: Supplementary file 1 [file plants-11-03370-s001.zip › plants-2039485-supplementary.pdf]

Effects of different fertilizer treatments on the yield, grain, flour, dough related traits and cookie quality of weak-gluten wheat

Supplementary materials and methods

1. Solvent retention capacity:

The retention capacity of flour in four solvents was measured according to AACC54-11 (Kweon et al 2011). Four reagents were prepared, including deionized water, 50% (w/w) sucrose solution, 5% (w/w) sodium carbonate solution, and 5% (w/w) lactic acid solution.

a. Four 50-ml centrifuge tubes with known weight were placed on the test tube rack, followed by the addition of 5 g weak-gluten wheat flour to each tube.

b. The four centrifuge tubes were respectively added with 25 g of each prepared reagent. The centrifuge tubes were covered and shaken for 5 s, followed by shaking at 5, 10, 15 and 20 min, and then centrifugation at 1000 g for 15 min.

c. After centrifugation, the suspension was poured off gently. Then, the test tubes were inverted on absorbent paper for 10 min. The test tubes were covered and weighed again.

The solvent retention capacity (SRC) was calculated according to the following formula:

SRC (%) = [glue weight / flour weight * 86 / (100 - flour moisture) - 1] *100

2. Making of two type of cookies:

Chinese crisp biscuits were made according to Chinese standard SB/T10141-93 (Standardization Administration 1993b), which mainly includes the following eleven steps:

1. First, 85.5 g sucrose was dissolved into 15 ml water. After heating, the sucrose solution was cooled to about 30 °C, followed by the addition of 13.8 g caramel.

2. About 45 g shortening and 6 g cream were heated and melt together, then the

mixture was cooled to about 30 ℃, followed by the addition of 0.012 g citric acid thereto.

3. About 0.21 g of baking soda was dissolved into 5 ml cold water, and 0.9 g of sodium chloride and 0.9 g of ammonium bicarbonate were dissolved together in the baking soda solution.

4. The ingredients obtained in the first step and the second step were mixed, and stirred with Horbot mixer at low speed for 10s, followed by the addition of 50 g of eggs and stirring.

5. The solution obtained in the third step was added to the mixture from the fourth step, then they were mixed at low speed.

6. All accessories (except for milk powder) were added to the mixture from the fifth and stirred for 1 min.

7. About13.8 g of milk powder and 300 g of flour were mixed evenly in advance, and then the above-mixed accessories were added to make dough, which was let to stand still for 1 min to make the temperature reach 22 °C.

8. The dough was taken out of the dough mixer and let to rest for 5–10 min. When kneading the dough by hand, if the hand felt sticky, and there were clear hand marks on the dough, and when pulling the dough, if the operator felt cohesive and extensible, the dough was considered to be ready for the making of Chinese crispy cookies.

9. The dough was cut into small pieces of 2.5–3 cm thickness.

10. The small dough was made into the shape of Chinese biscuits.

11. The dough was baked. The baking temperature was 200 °C and the time was about 9 min. Notably, the specific baking time depends on the color of the cookies.

American cookies were made according to standard AACC10-52 of America (Gaines CS 1986). The main steps for American cookie making are as follows.

1. Sucrose, skimmed milk powder and baking soda were added to the shortening after mixing and stirring them for eight times. The mixture was put into the mixing bowl and mixed at low speed for one minute, followed by twice mixing at high speed for 30 seconds, and then allowed to stand still. Finally, 37.6 g of the stirred mixture was obtained.

2. The mixture obtained in the first step was poured into a dough mixing bowl, followed by the addition of 4 ml of solution A, 2 ml of solution B and an appropriate amount of water, and stirring for 3 min.

3. The flour was put into the kneading bowl, and mixed with the mixture from step 2 to form a dough. Then, the dough was scrapted out of the kneading bowl.

4. The dough was cut into two equal parts with a small spatula, and rolled to the required thickness with a dough roller on the mold, and then the excess dough was cut off. The obtained dough was immediately put into a 205 °C oven for 11 min.

5. The biscuits were taken out from the oven, and the diameter and thickness of the biscuits were measured after cooling for about 30 min.

References

1. Gaines CS. Baking quality of cookie flour: micro method 10-52. 1986.

2. Kweon M，Slade L，Levine H et al．Solvent retention capacity (SRC) testing of wheat flour: principles and value in predicting flour functionality in different wheat-based food processes and in wheat breeding: a review．Cereal Chem. 2011，88: 537-552.

3. Standardization Administration. National standards of the People’s Republic of China: Wheat flour for crispy biscuit (SB/T10141-93). 1993b.
